# CPET Characteristics in HFrEF, HFpEF and COPD

**DOI:** 10.3390/jcm15145580

**Published:** 2026-07-16

**Authors:** Yuqing Zhou, Guimei Wang, Xiaohong Wu

**Affiliations:** Department of Respirology, Sir Run Run Shaw Hospital, School of Medicine, Zhejiang University, Hangzhou 310016, China; 3321042@zju.edu.cn (Y.Z.);

**Keywords:** cardiopulmonary exercise testing, heart failure with reduced ejection fraction, heart failure with preserved ejection fraction, chronic obstructive pulmonary disease, differential diagnosis

## Abstract

Chronic obstructive pulmonary disease (COPD) frequently occurs alongside heart failure (HF), including heart failure with reduced ejection fraction (HFrEF) and heart failure with preserved ejection fraction (HFpEF), creating a diagnostic dilemma due to overlapping clinical manifestations such as exertional dyspnea and reduced exercise tolerance. Static diagnostic modalities are often insufficient to distinguish these entities or their overlapping syndromes (HFrEF-COPD and HFpEF-COPD). Cardiopulmonary exercise testing (CPET) uniquely provides a dynamic, integrated assessment of the respiratory, cardiovascular, and peripheral muscle systems during incremental exercise, enabling quantitative characterization of the pathophysiological mechanisms underlying exercise limitation. This review comprehensively summarizes the characteristic CPET profiles of isolated HFrEF, isolated HFpEF, isolated COPD, HFrEF-COPD, and HFpEF-COPD. Integration of CPET parameters with pulmonary function testing results in a comprehensive assessment of the cardiopulmonary system, facilitating accurate diagnosis and prognostic stratification in this complex patient population. Finally, we address current limitations in standardization and outline future directions for advancing precision medicine in this complex clinical landscape.

## 1. Introduction

Chronic obstructive pulmonary disease (COPD) and heart failure (HF) are leading causes of global morbidity and mortality, with a well-documented bidirectional epidemiological association [1]. COPD patients have a 2–3-fold greater risk of developing HF, and the presence of HF in COPD patients elevates all-cause mortality by 50% [2]. Epidemiological data indicate that the prevalence of HF among COPD patients ranges from 7% to 42%, while the prevalence of COPD among HF patients is between 13.0% and 39.0% [3]. This cardiopulmonary overlap syndrome presents considerable challenges for clinical management, as both conditions manifest with dyspnea and exercise intolerance, rendering clinical history and physical examination often insufficient for accurate differentiation [4]. The pathophysiological mechanisms underlying heart failure with preserved ejection fraction (HFpEF) differ from those of heart failure with reduced ejection fraction (HFrEF), and the addition of COPD-related airflow obstruction further complicates exercise limitation, making comprehensive cardiorespiratory assessment essential [5].

Notably, COPD is more commonly associated with HFpEF than with HFrEF or heart failure with mid-range ejection fraction (HFmrEF) in clinical cohorts, with respective proportions of 16%, 11%, and 12% [6]. The clinical impact of this comorbidity is subtype-dependent: HFpEF-COPD is primarily associated with exacerbations of respiratory symptoms, whereas HFrEF-COPD correlates with a higher incidence of cardiovascular events and hospitalizations [7,8]. Given the paucity of effective pharmacotherapies for HFpEF, accurate differentiation between HFrEF-COPD and HFpEF-COPD is not only diagnostically critical but also essential for optimizing treatment and reducing the risk of COPD exacerbations [6].

Unlike static tests, CPET evaluates the integrated response of the cardiopulmonary system to physiological stress, uncovering dynamic abnormalities such as dynamic hyperinflation, ventilatory inefficiency, and chronotropic incompetence [7]. The European Society of Cardiology (ESC) and the American Heart Association (AHA) have emphasized CPET′s indispensable role in identifying the etiology of unexplained dyspnea, assessing functional impairment severity, and stratifying prognostic risk [4]. Recent years have witnessed extensive investigation into CPET parameter characteristics among patients with heart failure complicated by COPD, providing robust evidence that can guide clinical practice [9,10,11,12]. This narrative review focuses on the characteristics of CPET among patients with heart failure and COPD. We sorted out core CPET phenotypic characteristics, pathophysiological mechanisms and clinical application evidence, and summarized the existing limitations and future research directions.

## 2. Pathophysiological Mechanisms

### 2.1. Isolated HFrEF, HFpEF, and COPD

Isolated HFrEF is characterized by impaired left ventricular systolic function, leading to a reduced stroke volume and cardiac output, directly limiting the delivery of working muscles during exercise [13]. The relationship between cardiac output and oxygen uptake during exercise can be described by the Fick principle: VO_2_ = cardiac output × arteriovenous oxygen difference. In HFrEF, both components of this equation are compromised, resulting from impaired contractility, abnormal ventricular remodeling, and often concomitant mitral regurgitation. Additionally, endothelial dysfunction and abnormal skeletal muscle metabolism contribute to reductions in peripheral oxygen extraction, further limiting peak VO_2_ [14]. Elevated left ventricular filling pressure is transmitted retrograde to the pulmonary circulation, causing pulmonary congestion, interstitial edema, and ventilation perfusion (V/Q) mismatch. This results in expanded physiological dead space and enhanced chemoreceptor sensitivity, manifesting as an elevated VE/VCO_2_ slope, which is an indicator of ventilatory efficiency (defined as the rate of change in minute ventilation versus carbon dioxide production) [15]. Right ventricular (RV) dysfunction, commonly observed in advanced HFrEF, further exacerbates V/Q mismatch and correlates strongly with VE/VCO_2_ slope elevation, predicting adverse outcomes including RV failure following mechanical circulatory support [16].

Isolated HFpEF (LVEF ≥ 50%) is defined by abnormal left ventricular diastolic function, resulting in elevated left heart filling pressures at rest and/or during exercise, which causes pulmonary congestion and exertional dyspnea [16], a core pathological feature that further induces bronchial mucosal edema and increases airway resistance in afflicted patients [17].

COPD is characterized by persistent airflow limitation, airway inflammation, and parenchymal destruction. During exercise, dynamic hyperinflation develops as incomplete expiration leads to air trapping and an increased end-expiratory lung volume. This results in flattened diaphragms operating at unfavorable lengths, increased work required for breathing, and reduced inspiratory capacity [18]. The mechanical limitation to ventilation manifests as reduced breathing reserve and early attainment of maximal voluntary ventilation during CPET. Emphysematous destruction of alveolar walls leads to loss of alveolar capillary beds and impaired gas exchange. The diffusion capacity for carbon monoxide (DLCO) is reduced in COPD, reflecting both destruction of alveolar surface area and reduced pulmonary capillary blood volume. This impairment limits oxygen transfer during exercise, contributing to exercise-induced desaturation. Chronic hypoxemia triggers hypoxic pulmonary vasoconstriction, smooth muscle hypertrophy, and intimal fibrosis in pulmonary arteries, resulting in pulmonary hypertension that increases right ventricular afterload.

Patients with COPD exhibit elevated VE/VCO_2_ slopes due to increased physiological dead space and a heightened ventilatory drive [19]. The V/Q mismatch, particularly in emphysema with areas of low V/Q and a large amount of dead space, necessitates increased minute ventilation relative to metabolic CO_2_ production. Exercise oscillatory ventilation may also be observed, reflecting unstable ventilatory control [11].

### 2.2. Reciprocal Interplay Among COPD, HFrEF and HFpEF

COPD exerts multiple adverse effects on cardiovascular function through mechanical, hypoxic, autonomic dysfunction and systemic inflammation pathways [9,20,21,22], with differential impacts on HFrEF and HFpEF. Dynamic hyperinflation in COPD increases intrathoracic pressure and reduces venous return, impairing biventricular preload and further reducing stroke volume in already compromised HFrEF patients [23,24,25,26,27]. Conversely, HF-induced pulmonary congestion exacerbates airway obstruction in COPD patients through bronchial mucosal edema, forming a vicious cycle of mutual aggravation [17]. Recent studies have confirmed that there is an inverse linear correlation between FEV_1_ reduction and NT-proBNP elevation as well as an increase in pulmonary pressure in stable HF patients, suggesting there is a volume-dependent obstructive component induced by the interaction of the two diseases [28].

Among HFpEF patients, COPD-induced lung hyperinflation synergizes with HFpEF-related pulmonary congestion to causing a dual increase in airway resistance. Recent studies using cardiac magnetic resonance (CMR) have revealed that HFpEF patients with comorbid COPD have more severe myocardial fibrosis, systemic large artery stiffening, and LV remodeling than isolated HFpEF patients [29], a difference that may be related to the synergistic effect of systemic inflammation in COPD and hemodynamic overload in HFpEF, and this pathological change is closely associated with abnormal ventilatory efficiency parameters in CPET [30].

Alveolar destruction (COPD) and pulmonary capillary congestion (heart failure) synergistically disrupt V/Q matching, increasing both the VE/VCO_2_ slope and VE intercept while drastically shrinking BR. The combined injury to the alveolar–capillary membrane also causes a profound DLCO reduction, serving as a core pathological driver of ventilatory inefficiency and elevated VE/VCO_2_ in heart failure–COPD overlap patients.

## 3. Core Cardiopulmonary Exercise Testing Parameters

### 3.1. Ventilatory Efficiency Parameters

Ventilatory efficiency constitutes one of the core parameters for assessing exercise function in patients with cardiopulmonary diseases, primarily comprising the VE/VCO_2_ slope and VE intercept. The VE/VCO_2_ slope, defined as the ratio of minute ventilation (VE) to carbon dioxide production (VCO_2_) during exercise, reflects the matching between alveolar ventilation and gas exchange. Under normal physiological conditions, alveolar ventilation maintains precise coordination with CO_2_ production as exercise intensity increases, with the VE/VCO_2_ slope remaining relatively constant [31].

The VE intercept is attracting increasing research attention and has demonstrated unique value in differentiating COPD from HFpEF. This parameter reflects the degree of alveolar dead space ventilation, being substantially elevated in COPD patients while remaining relatively low in HFpEF patients.

Breathing reserve (BR) reflects the relationship between ventilatory capacity and ventilatory demand during exercise. It is calculated as the difference between maximal voluntary ventilation (MVV) and peak exercise minute ventilation (VEpeak), expressed either as an absolute value or as a ratio. Breathing reserve is considered normal when it exceeds 20% of MVV or 15 L/min in males [32].

### 3.2. Gas Exchange Parameters

Peak oxygen uptake (Peak VO_2_), i.e., the maximal capacity for oxygen delivery and utilization during exercise, is one of the most extensively studied CPET parameters.

Anaerobic threshold (AT) constitutes the highest oxygen uptake (VO_2_) at which lactate accumulation does not exhibit a sustained increase during incremental exercise, signifying the onset of anaerobic metabolism. In healthy individuals, the AT typically occurs at 40–80% of maximal oxygen uptake (VO_2_max), most commonly at 50–60% of VO_2_max [32].

The oxygen uptake efficiency slope (OUES) is an increasingly recognized CPET variable that reflects the efficiency of oxygen utilization during exercise. OUES is derived from the logarithmic correlation between oxygen uptake (VO_2_) and minute ventilation (VE) across exercise stages, and its calculation does not depend on achievement of maximal exercise intensity [33]. As such, this parameter is especially suitable for patients who cannot perform a maximal exercise test.

### 3.3. Hemodynamic Parameters

Circulatory power (CP) is a composite index evaluating cardiovascular system function, defined as the product of peak oxygen uptake and peak systolic blood pressure (Peak VO_2_ × Peak SBP), expressed in mmHg·mL·kg^−1^·min^−1^. This parameter comprehensively assesses cardiovascular oxygen delivery capacity and blood pressure regulation function, holding significant prognostic value in regard to HFpEF-COPD patients [34].

Oxygen pulse (O_2_ pulse) is defined as the VO_2_/heart rate, and it is expressed as mL/kg/beat. It indicates the maximum stroke volume.

### 3.4. Integrated Application with Pulmonary Function Testing

Combined assessment of pulmonary function indices and CPET parameters enhances differential diagnostic accuracy. HF-COPD patients with an FEV_1_ < 1.6 L exhibit worse exercise tolerance and have a higher risk of hospitalization [34]. FEV_1_ is negatively correlated with VO_2_/Work Rate (r = −0.735, *p* < 0.001), indicating that more severe airway obstruction is associated with lower exercise efficiency [34]. An FEV_1_/FVC ratio < 0.7 supports COPD diagnosis, while the correlation between absolute FEV_1_ value and exercise tolerance helps assess disease severity [35]. Therefore, the integrated application of pulmonary function testing and CPET enables comprehensive evaluation of the cardiopulmonary system from both cardiac and pulmonary perspectives.

## 4. Characteristic CPET Profiles of Isolated HFrEF, HFpEF, and COPD

Distinguishing HFrEF, HFpEF and COPD accurately requires a full grasp of their respective CPET features, which have been documented in a large body of comparative studies [36].

Isolated HFrEF patients exhibit significantly reduced exercise tolerance and impaired ventilatory efficiency, manifesting as a markedly decreased peak VO_2_ and oxygen pulse (O_2_ pulse), an elevated VE/VCO_2_ slope, and reduced oxygen uptake efficiency slope (OUES) [34,36,37]. In the Weber–Janicki grading system, peak VO_2_ is used to classify exercise tolerance in HFrEF patients, with lower values indicating worse cardiac function [38]. A normal VE/VCO_2_ slope is usually below 30, while for HFrEF patients this parameter often exceeds 35, suggesting ventilation–perfusion mismatch and increased dead space ventilation due to pulmonary edema [36,37]. Furthermore, HFrEF patients also have a significantly reduced OUES, reflecting the decline in systematic oxygen uptake efficiency during exercise [34].

HFrEF patients usually have a reduced AT due to impaired cardiac output augmentation and skeletal muscle metabolic abnormalities. Relative to HFrEF, patients with isolated HFpEF typically exhibit mild to moderate reductions in exercise tolerance, with most studies showing that HFpEF patients have a peak VO_2_ of between 12 and 16 mL/kg/min [36,37,39], and they exhibit a relatively preserved AT and OUES [37]. Studies have demonstrated that the VE/VCO_2_ slope in patients with heart failure with HFpEF typically ranges from 30 to 35, indicating a moderate degree of ventilatory inefficiency that is consistently less severe than that observed in patients with HfrEF [40,41]. Both HFpEF and HFrEF circulatory function parameters such as peak VO_2_ and O_2_P are significantly reduced in this condition, reflecting impaired stroke volume and cardiac output, with respiratory reserve typically preserved, a typical feature of cardiac exercise limitation [21,36]. Notably, these two indicators alone cannot reliably distinguish the two HF subtypes (Table 1 and Table 2).

The most prominent manifestation of COPD in CPET is ventilatory limitation driven by airflow obstruction and pulmonary hyperinflation. These patients exhibit markedly diminished BR, with ventilation approaching or peaking at maximal ventilatory capacity at exercise cessation, indicating severely depleted breathing reserve. Clinically, a BR cutoff of 30% and an AT corresponding to 40% of predicted peak VO_2_ are widely used to differentiate ventilatory limitation from cardiac limitation [42,43]. This represents the most critical distinction between COPD and heart failure in CPET [43,44,45]. In mild-to-moderate COPD, the VE/VCO_2_ slope is elevated; however, as the disease progresses to advanced stages, the slope paradoxically declines due to mechanical constraints and CO_2_ retention that blunt the ventilatory response [46]. A 2024 investigation further verified that impaired exercise ventilatory efficiency in individuals with COPD is closely linked to heightened mechanical restrictions, deteriorated pulmonary gas exchange, elevated dyspnea levels, and compromised exercise performance [46,47]. Notably, a rise in the ventilation intercept has been identified as the most reliable indicator for tracking the deterioration in exercise-related ventilatory inefficiency throughout all stages of COPD severity [46]. In COPD patients, peak VO_2_ is reduced because of mechanical constraints and deconditioning, while O_2_ pulse is often preserved [36,48]. AT and OUES values are markedly reduced due to ventilatory limitation, impaired gas exchange with increased dead space ventilation, and peripheral muscle dysfunction [49,50] (Table 1 and Table 2).

**Table 1:** Overview of study characteristics and key cardiopul1monary exercise testing (CPET) results.

Study	Design	*n*	Participants	Medication	CPET Parameters	Main Findings
Smith 2019 [36]	Cross-sectional observational comparative study	63	COPD vs. HFpEF vs. HFrEF (HFpEF: LVEF ≥ 50%; HFrEF: LVEF ≤ 40%)	COPD vs. HFpEF vs. HFrEF Bronchodilator 68% vs. 0 vs. 0 β-blocker 18% vs. 76% vs. 85%	COPD vs. HFpEF vs. HFrEF VE intercept, L/min: 3.32 ± 1.66 vs. 0.77 ± 1.23 vs. 1.28 ± 1.19; 2.64 L/min discriminated COPD from HF patients (AUC: 0.88, *p* < 0.01) VE/VCO_2_: 32 ± 7 vs. 32 ± 7 vs. 40 ± 9 Peak VO_2_: 17 ± 4 vs. 8 ± 2 vs. 9 ± 3	COPD had higher VE intercept than HFpEF/HFrEF (no HF subgroup difference); HFrEF showed elevated VE/VCO_2_ slope vs. COPD and HFpEF. VE intercept ≥2.64 L/min distinguished COPD from HF subtypes (AUC = 0.88, *p* < 0.01), while VE/VCO_2_ slope lacked discriminative value.
Campos 2024 [51]	Retrospective cross-sectional study	38	COPD vs. HF (HF: LVEF %, 45 ± 16)	COPD vs. HF β-blocker: 0 vs. 95% LABA: 100% vs. 0 LAMA: 26% vs. 0	COPD vs. HF VE/MVV (%), 0.95 ± 0.2 vs. 0.5 ± 0.1 VE/VCO_2_ slope: 27.2 ± 1.4 vs. 33.1 ± 5.7 VE intercept: 5.3 ± 1.9 vs. 1.7 ± 3.6	VE/VCO_2_ slope and intercept were significantly different for COPD and CHF (27.2 ± 1.4 vs. 33.1 ± 5.7 and 5.3 ± 1.9 vs. 1.7 ± 3.6, *p* < 0.05 for both).
Barron 2016 [43]	Prospectively observational study	199	COPD vs. HFrEF vs. Healthy Adults (HFrEF: LVEF % 35.3 ± 9.4)	COPD vs. HFrEF vs. Healthy Adults β-Blocker 3% vs. 86% vs. 17%	COPD vs. HFrEF vs. Healthy Adults Peak VO_2_, mL/min/kg: 16.7 ± 0.9 vs. 16.5 ± 0.7 vs. 24.4 ± 0.4 AT, mL/min: 983 ± 60 vs. 941 ± 39 vs. 1157 ± 22 OUES: 2.11 ± 0.09 vs. 1.50 ± 0.07 vs. 2.27 ± 0.04 O_2_ pulse, mL/beat: 10.7 ± 0.5 vs. 12.1 ± 0.4 vs. 13.8 ± 0.2 VE/VCO_2_ slope: 33.6 ± 1.1 vs. 36.0 ± 0.8 vs. 26.5 ± 0.5 BR, %: 9.3 ± 3.0 vs. 42.6 ± 2.3 vs. 46.8 ± 1.2 CP, mmHg mL kg^−1^ min^−1^: 259.3 ± 20.7 vs. 196.6 ± 15.6 vs. 398.9 ± 8.5	Breathing reserve (AUC: 0.91) and OUES (AUC: 0.87) had the greatest ability to discriminate between COPD and HFrEF. VO_2_ at the AT did not discriminate (AUC for AT as percent predicted peak VO_2_: 0.56).
Nadruz Jr 2017 [37]	Longitudinal observational study	825	HFrEF vs. HFpEF (HFpEF: LVEF ≥ 50%; HFrEF: LVEF ≤ 40%)	HFrEF vs. HFpEF β-Blocker: 90% vs. 69%	HFrEF vs. HFpEF VE/VCO_2_ slope: 34.5 ± 9.2 vs. 30.3 ± 6.7 Peak VO_2_, mL/min/kg: 14.3 ± 5.2 vs. 17.4 ± 7.8	Peak VO_2_ and VE/VCO_2_ slope offer independent incremental prognosis for HFpEF endpoints; their predictive performance is stronger in HFpEF than HFrEF.
Goulart et al. 2020 [34]	Cross-sectional study	40	COPD-HFrEF (LVEF% 39 ± 8)	β-Blocker (100%)	Peak VO_2_, mL/min/kg: 12 ± 3 VE/VCO_2_ slope: 38 ± 10 O_2_ pulse (mL.bpm^−1^): 10 ± 5 CP (mmHg.mLO_2_.min^−1^): 2045 ± 727 OUES 1.3 ± 0.3	OUES < 1.3 and CP < 2383 serve as thresholds for HF-COPD patients with FEV_1_ < 1.6 L; OUES < 1.3, CP < 2116 and VE/VCO_2_ slope > 38 are cutoffs for HF-COPD with LVEF < 39% (*n* = 19); In Kaplan–Meier analysis: LVEF < 39% and FEV_1_ < 1.6, VE/VCO_2_ > 38, OUES < 1.3 and CP < 2383 were prognostic indicators for hospitalization in COPD-HF patients.
Guazzi 2010 [52]	Matched case–control clinical study	138	HF vs. HF+COPD (HF: LVEF% 34.8 ± 9.7; HF+COPD: 33.8 ± 11.9)	β-Blocker (HF:88% vs. HF+COPD: 89.8%)	HF vs. HF+COPD Peak VO_2_: 16.3 ± 4.3 vs. 12.1 ± 4.3 VE/VCO_2_ slope: 33.3 ± 6.6 vs. 42.7 ± 7.4 VE/MVV, %: 59.4 ± 15.0 vs. 66.4 ± 14.5	HF+COPD group: Peak VO_2_, maximal HR, HRR, and 6MWT distance were significantly lower, whereas the VE/VCO_2_ slope, frequency of EOV, VE/MVV, and peak dyspnea were significantly higher.
Santos 2022 [49]	Longitudinal observational study	124	HF vs. COPD vs. HF+COPD (HF: LVEF ≤ 50%)	HF vs. COPD vs. HF+COPD β-blocker:46 vs. 0 vs. 24 Bronchodilator: 1 vs. 53 vs. 21	HF vs. COPD vs. HF+COPD VO_2_, mL·min: 1011 ± 414 vs. 859 ± 228 vs. 806 ± 300 O_2_ pulse, mL/beat: 8.5 ± 3 vs. 7.1 ± 2 vs. 7.7 ± 3 CP, mmHg mL kg^−1^ min^−1^ 2439 ± 857 vs. 2451 ± 741 vs. 1987 ± 685 VE intercept, L/min:1.0 ± 2 vs. 3.0 ± 3 vs. 0.7 ± 2	HF+COPD had the greatest impairment with cardiorespiratory fitness, expressed by lower values in key CPET variables. WR and VO_2_ peak significantly lower in HF+COPD than HF; COPD group demonstrated a significantly lower O_2_ pulse compared to the HF group; COPD group had higher VE intercept compared to the other groups.
Goulart 2020 [53]	Cross-sectional study	22	HF vs. COPD+HF (HF: LVEF < 50%)	HF vs. COPD+ HF β-blocker:100% vs. 100%	WR, W: 64 ± 23 vs. 83.7 ± 34 VO_2_, mL/min: 890 ± 333 vs. 1017 ± 337	COPD coexistence reduces cerebral oxygenation and exacerbates muscle deoxygenation and exertional dyspnea in HF patients.
Santos 2021 [13]	Cross-sectional study	46	HF vs. HF+COPD (HF: LVEF%: 40 ± 5; HF+COPD:38 ± 7)	HF vs. HF+COPD β-blocker:96% vs. 96% LAMA:0 vs. 43% LABA: 0 vs. 34%	HF vs. HF+COPD O_2_ pulse, mL/beat: 10 ± 3 vs. 5 ± 2 CP, mmHgm kg^−1^ min^−1^: 2730 ± 768 vs. 2051 ± 735 VO_2_, mL·min: 1109 ± 349 vs. 847 ± 307	HF+COPD compared to HF: Lower work rate, VO_2_ peak, O_2_ pulse, rate pressure product (RPP), CP and ventilatory power (VP).
Boulet 2025 [54]	Retrospective cross-sectional study	1994	COPD vs. HF vs. COPD-HF (LVEF unclear)	COPD– vs. HF vs. COPD-HF β-blocker: 13% vs. 53% vs. 71%	COPD– vs. HF vs. COPD-HF Peak VO_2_ (mL/kg/min): [23.62 (23.06, 24.19)] vs. [22.44 (21.85, 23.02)] vs. [19.93 (18.60, 21.27)] * VE/VCO_2_ slope: [29.67 (29.25, 30.09)] vs. [33.91 (33.50, 34.33)] vs. [36.73 (35.78, 37.68)] *	COPD-HF had higher VE/VCO_2_ slope (36.73, 95%CI 35.78–37.68 vs. 33.91, 95%CI 33.50–34.33, *p* < 0.0001) versus HF alone and the lowest peak VO_2_ (19.93 mL/kg/min, 95%CI 18.60–21.27) versus other groups.
Cherneva 2020 [55]	Cross-sectional study	68	non-severe COPD vs. COPD+ HFpEF (FEV_1_ > 50%, LVEF > 50%)	COPD vs. COPD+ HFpEF β-blocker: 0 vs. 0 LABA: 48% vs. 28% LAMA: 52% vs. 40%	COPD vs. COPD+ HFpEF Peak VO_2_, mL kg^−1^ min^−1^: [23.78 (21.98–24.97)] vs. [18.31 (15.72–19.56)] VE/VCO_2_ slope: [26.58 (23.81–30.68)] vs. [31.45 (27.24–33.68)] HRR at 1 min, bpm: [16.5 (14–18)] vs. [9.5 (6–10)]	AD is more common in the HFpEF group. VO_2_ peak, O_2_ pulse, ventilatory efficiency and HRR correlate to masked HFpEF HRR as the only independent predictor of masked HFpEF—(OR 10.28; 95% CI (3.55–29.80)).
Cherneva 2021 [56]	Retrospective cross-sectional study	104	COPD vs. COPD+ HfpEF ^†^ (COPD with LVDD−, LVDD+, RVDD−, RVDD+) (FEV_1_ > 50%, LVEF < 50%)	Not reported	LVDD−, LVDD+; RVDD−, RVDD+ Peak VO_2_, mL/min/kg: [14.30(12.6–16.15)] vs. [13.90 (12.67–15.7)]; [14.30 (12.6–16.15)] vs. [13.40(15.77–12.55)] O_2_ pulse mL/min/kg: [9.80 (9.5–12.2)] vs. [7.90 (6.15–9.32)]; [9.51 (9.02–13.1)] vs. [7.92(6.27–9.84)] VE/VCO_2_ slope: [34.08 (33.98–36.72)] vs. [36.93 (34.19–38.74)]; [34.11 (33.78–36.89)] vs. [36.98 (34.26–38.91)]	COPD with stress-induced diastolic dysfunction (both LVDD and RVDD) achieve lower load, VO_2_ and O_2_-pulse and perform with significantly higher VE/VCO_2_ slopes.
Arbex 2016 [57]	Prospective cross-sectional observational comparative study	98	COPD+ HFrEF vs. COPD (HF: LVEF ≤ 45%)	COPD vs. COPD+ HFrEF β-blockers (3.5% vs. 85.4%) LABA (100% vs. 85.5%) LAMA (75.4% vs. 69.4%)	COPD vs. Overlap Peak VO_2_, mL/minute 1361 ± 450 vs. 1048 ± 287 VE/MVV 0.84 ± 0.18 vs. 0.74 ± 0.19 VE/VCO_2_ slope: 28.9 ± 6.0 vs. 36.0 ± 6.6 VE intercept, L/minute: 6.0 ± 2.9 vs. 2.9 ± 2.7 Peak PETCO_2_, mmHg: 38 ± 7 vs. 32 ± 6	Overlap group: lower peak workload, peak VO_2_ and VE intercept, with impaired ventilatory efficiency (higher VE/VCO_2_ ratio and slope) yet preserved ventilatory reserve (lower VE/MVV). Higher AUCs to discriminate overlap from COPD: VE intercept ≤ 3.5 L/minute plus VE/VCO_2_ Slope ≥ 34 or peak VE/VCO_2_ ratio ≥ 37 with peak PETCO_2_ ≤ 31 mmHg Similar results were found contrasting FEV_1_-matched COPD and overlap patients.
Urrutia 2025 [58]	Cross-sectional comparative study	65	HFrEF vs. HFrEF+COPD (HFrEF: LVEF ≤ 40%)	HFrEF vs. HFrEF+COPD β-blockers:95.8% vs. 100%	BR at max. exercise (%): HFrEF:30 (25.3–34.9) vs. HFrEF+COPD 12 (2–35)	HFrEF+COPD group: Patients with HFrEF-COPD presented significantly lower BR and a higher VE/VCO_2_ slope.
Apostolo 2015 [59]	Retrospective cross-sectional study	309	HFrEF vs. HFrEF-COPD vs. COPD (HF: LVEF < 40%)	Not reported	HFrEF vs. HFrEF-COPD vs. COPD VO_2_ peak, mL/kg/min: 15.5 ± 5.1 vs. 14.8 ± 4.3 vs. 18.6 ± 4.9 VE/VCO_2_ slope: 32 ± 7, vs. 31 ± 7 vs. 31 ± 6 VE intercept, L/minute: 3.0 ± 2.6 vs. 4.8 ± 2.4 vs. 5.9 ± 3.0	A high VE intercept (≥4.07 L/min) indicates patients with high probability of having COPD with or without HF (AUC = 0.76). No slope differences were observed among HF, HF-COPD and COPD. VE intercept was higher in HF-COPD and COPD compared to HF (*p* < 0.01).

Values are mean ± SD, *n* (%), or median (interquartile range). Abbreviations: AD, autonomic dysfunction; AUC, Area Under the Curve; bpm, beats per minute; BR, Breathing reserve; CP, circulatory power; COPD, chronic obstructive pulmonary disease; FEV_1_, forced expiratory volume in 1 s; HF, heart failure; HFpEF, heart failure with preserved ejection fraction; HFrEF, Heart failure with reduced ejection fraction; HR, heart rate; HRR, heart rate recovery; LABA, long-acting β_2_-agonists; LAMA, long-acting muscarinic antagonist; LVEF, left ventricular ejection fraction; LVDD, left ventricular diastolic dysfunction; O_2_ pulse, oxygen pulse; OUES, oxygen uptake efficiency slope; RVDD, right ventricular diastolic dysfunction; VP, ventilatory power; VE, minute ventilation; VE/VCO_2_ slope, ventilatory efficiency; VO_2_, oxygen uptake/oxygen consumption. * CPET outcomes adjusted for age, sex, and β-blocker use; ^†^ Patients were analyzed separately in two stratifications: grouped by the presence of stress-induced LVDD (COPD without stress LVDD/COPD with stress LVDD), and grouped by stress-induced RVDD (COPD without stress RVDD/COPD with stress RVDD).

**Table 2:** Comparison of core CPET characteristics.

CPET Parameter	Isolated HFrEF	Isolated HFpEF	Isolated COPD	HFrEF-COPD Overlap	HFpEF-COPD Overlap	Note
Peak VO_2_	Greatly reduced	Moderately reduced	Reduced	Greatly reduced	Greatly reduced	Prognostic indicator.
VE/VCO_2_ slope	Elevated	Elevated	Elevated	Elevated	Elevated	Prognostic indicator.
VE intercept	Low	Low	High	High	High	VE intercept ≥ 2.64 L/min distinguished COPD from HF subtypes (AUC = 0.88)
BR	Preserved	Preserved	Markedly reduced	Markedly reduced	Markedly reduced	Breathing reserve (AUC: 0.91) had the greatest ability to discriminate between COPD and HFrEF
OUES	Reduced	Reduced	Mildly reduced in GOLD I-II stage	Markedly reduced	Markedly reduced	OUES (AUC: 0.87) had the greatest ability to discriminate between COPD and HFrEF.
O_2_ pulse	Greatly reduced	Greatly reduced	Near-normal/Reduced	Greatly reduced	Greatly reduced	NA
CP	Decreased	Moderately decreased	Near-normal/Reduced	Lowest	Reduced	Prognostic indicator.
Dominant exercise limitation	Circulatory (systolic cardiac dysfunction)	Circulatory (diastolic cardiac dysfunction)	Ventilatory (airflow obstruction and hyperinflation)	Combined circulatory + ventilatory limitation	Combined circulatory + ventilatory limitation	NA

Smith et al. (2019) [36] reported distinct patterns of VE/VCO_2_ slope and VE intercept across HFpEF, HFrEF, and COPD patients. HFrEF patients exhibited the highest VE/VCO_2_ slope (40 ± 9) but a relatively low VE intercept (1.28 ± 1.19 L/min), and HFpEF patients showed a moderate VE/VCO_2_ slope (32 ± 7) with the lowest VE intercept (0.77 ± 1.23 L/min); whereas COPD patients demonstrated a moderate VE/VCO_2_ slope (32 ± 7) but the highest VE intercept (3.32 ± 1.66 L/min) among the three groups. This markedly increased VE intercept in COPD patients indicates that VE intercept, rather than VE/VCO_2_ slope, may distinguish COPD from both HFpEF and HFrEF. A study by Barron et al., that compared the discriminative performance of CPET indices among patients with cardiac and respiratory diseases, revealed that BR and OUES were the most effective CPET parameters for differentiating HF from COPD, whereas the anaerobic threshold (AT) lacked discriminative value in these patient cohorts [43] (Table 1 and Table 2).

## 5. CPET Profiles of HFrEF-COPD and HFpEF-COPD Overlap Syndromes

### 5.1. HFrEF-COPD Overlap Syndrome

In this review, HFrEF-COPD overlap syndrome is defined as the coexistence of reduced or mid-range left ventricular ejection fraction (LVEF < 50%) and obstructive ventilatory defects confirmed by spirometry [59], and its CPET profile integrates the characteristics of isolated HFrEF and COPD, showing a unique phenotypic signature resulting from the combined effect of systolic cardiac dysfunction and lung hyperinflation [59].

Patients exhibit markedly reduced peak VO_2_ (often below 14 mL/kg/min), reflecting the combined effects of impaired cardiac output and respiratory dysfunction [34,49]. Ventilatory efficiency is significantly impaired, with elevated VE/VCO_2_ slope typically exceeding 34, a commonly used cutoff for poor prognosis [60], indicating both ventilation–perfusion mismatch from pulmonary congestion and increased physiological dead space from airway obstruction [34,61], which also differs significantly between HFrEF-COPD patients and isolated HFrEF patients [58,59]. Notably, Apostolo et al. demonstrated that, regarding ventilatory efficiency parameters, the VE intercept was significantly higher in patients with HFrEF-COPD relative to those with isolated HFrEF (4.8 ± 2.4 vs. 3.0 ± 2.6 L/min), while no significant differences were found between the HFrEF–COPD group and the isolated COPD group (5.9 ± 3.0 L/min). Another finding showed that a VE intercept ≥ 4.07 L/min in HF patients should raise suspicion of COPD comorbidity and warrant further diagnostic investigations [59]. These findings suggest that the ventilatory limitation component is predominantly driven by COPD rather than systolic heart failure [57]. BR is notably reduced, approaching values seen in isolated COPD, which distinguish HFrEF-COPD overlap from isolated HFrEF, where BR is typically preserved [43,57,62]. Additionally, Goulart et al. showed that OUES < 1.3, CP < 2116 mmHg·mLO_2_·kg^−1^·min^−1^ and VE/VCO_2_ slope > 38 are effective thresholds with which to screen HF-COPD patients with LVEF < 39% (moderate-to-severe systolic heart failure) [34].

In terms of pulmonary function parameters, Apostolo et al. [59] reported that the average predicted FEV_1_ percentage in patients with HFrEF-COPD was 55.4 ± 14.9, which was almost identical to that in patients with isolated COPD (55.4 ± 14.8). Forced vital capacity (FVC) was reduced in the HFrEF-COPD, isolated COPD, and isolated HFrEF groups, but the reduction was significantly milder in the isolated HFrEF group than in the HFrEF-COPD group (Table 1 and Table 2).

### 5.2. HFpEF-COPD Overlap Syndrome

HFpEF-COPD overlap syndrome is diagnosed in patients with LVEF ≥ 50%, clinical symptoms of HF, elevated left heart filling pressures at rest and/or during exercise, and concomitant obstructive ventilatory defects [13], and its CPET characteristics are a fusion of diastolic cardiac dysfunction and COPD-induced increased dead space ventilation [36].

Regarding gas exchange parameters, HFpEF-COPD patients demonstrate characteristic patterns [36,63]. First, both peak VO_2_ and oxygen uptake at the anaerobic threshold (VO_2_ at AT) are significantly reduced [9], reflecting dual obstacles in oxygen delivery and utilization. Second, O_2_ pulse is reduced [56]. O_2_ pulse reduction may result from an insufficient increased in stroke volume during exercise, or impaired peripheral oxygen extraction due to skeletal myopathy and exercise deconditioning in patients with heart failure and COPD. In most cases, it is caused by the combination of the above two factors [64,65]. Ventilatory parameter abnormalities represent another significant feature in HFpEF-COPD patients. HFpEF-COPD patients display lower VE at identical exercise workloads, along with reduced peak VO_2_, O_2_ pulse, HRR and impaired ventilatory efficiency, as well as significantly elevated VE/VCO_2_ slopes relative to patients with isolated non-severe COPD. Multivariate regression analysis showed HRR as the only independent predictor of masked HFpEF (OR 10.28; 95% CI (3.55–29.80)) [55].

The VE intercept demonstrates unique value in differentiating COPD from HF. Research confirms that the VE intercept is significantly higher in COPD patients than in HFpEF patients (3.32 ± 1.66 L/min vs. 0.77 ± 1.23 L/min, *p* < 0.01), reflecting the greater pulmonary dead space ventilation proportion in COPD patients [36]. A VE intercept threshold of 2.64 L/min yields an AUC of 0.88 with a specificity up to 95% for distinguishing COPD from heart failure. For populations consisting of COPD and HFpEF patients, researchers used ROC curve analysis to establish a VE intercept cutoff value of ≥1.82 L/min to identify individuals with a high likelihood of having COPD, with an AUC of 0.90 [36]. This finding holds important clinical significance for distinguishing cardiogenic from pulmonary causes of dyspnea in clinical practice.

OUES and VE/VCO_2_ slope together constitute important tools for assessing cardiopulmonary function in HF-COPD patients [34]. An OUES < 1.3 has been confirmed to be a sensitive indicator for predicting hospitalization in these patients, while VE/VCO_2_ slope > 38 suggests a poor prognosis. A condition wherein both these parameters are abnormal simultaneously suggests there is a severe dysfunction in both cardiopulmonary systems requiring aggressive intervention.

## 6. Prognostic Evaluation and Clinical Application

Multiple studies have confirmed the value of CPET parameters in predicting hospitalization risk among HF-COPD patients. In a follow-up study including 40 HF-COPD patients, Kaplan–Meier survival analyses demonstrated that the following CPET parameters can serve as robust predictors of hospitalization for HF-COPD patients (*p* ≤ 0.05): LVEF < 39% combined with FEV1 < 1.6 L, VE/VCO_2_ slope > 38, OUES < 1.3, and circulatory power < 2383 mmHg·mL·min^−1^ [34].

Peak VO_2_ and the VE/VCO_2_ slope are well-established prognostic markers for all-cause mortality. Studies have demonstrated that HF patients with a peak VO_2_ < 14 mL/kg/min [66] and VE/VCO_2_ slope > 34 face poor prognosis [67]. A 42-month follow-up cohort study [68] enrolling 126 patients with COPD demonstrated via Kaplan–Meier analysis that a VE/VCO_2_ slope ≥ 30, peak VE ≤ 25.7 L/min, and peak VO_2_ ≤ 13.8 mL O_2_·kg^−1^·min^−1^ are powerful independent predictors of all-cause mortality among individuals with COPD. These thresholds have been adopted by multiple international guidelines for guiding decisions regarding advanced therapies such as cardiac transplantation or left ventricular assist devices. For HF-COPD patients, given that COPD itself constitutes an important risk factor regarding mortality, the prognosis may be even worse, necessitating more aggressive comprehensive management [69].

## 7. Effects of β-Blockers on CPET

Distinct β-blocker subtypes have divergent impacts on CPET phenotypes in HF patients, an effect primarily driven by differential β_2_ receptor blockades [70]. Non-selective carvedilol suppresses excessive chemoreceptor activation and significantly lowers the VE/VCO_2_ slope during exercise, alleviating HF-related hyperventilation and improving patients’ self-reported dyspnea status. However, its concurrent β_2_ antagonism impairs alveolar epithelial fluid clearance and reduces the lungs’ diffusion capacity for carbon monoxide (DLCO) via depressed membrane conductance (Dm) [71,72]. This unfavorable pulmonary effect is amplified in individuals with pre-existing gas exchange impairment (DLCO <80% predicted) or concomitant chronic obstructive pulmonary disease (COPD), and under hypoxic conditions such as high altitude or air travel, carvedilol blunts compensatory ventilatory responses, further lowering arterial oxygen partial pressure and worsening exercise tolerance [73]. In contrast, highly β_1_-selective agents such as bisoprolol and nebivolol spare pulmonary β_2_ signaling, maintaining intact alveolar fluid transport and stable DLCO levels without interfering with ventilatory efficiency or hypoxic respiratory compensation [74]. Accordingly, HF patients receiving bisoprolol demonstrate marginally higher peak VO_2_ and O_2_ pulse values compared to those treated with carvedilol, particularly in cohorts with impaired baseline diffusion function [74]. Notably, concurrent cardioselective β-blocker administration does not counteract the exercise benefits of LAMA/LABA dual bronchodilation in COPD patients; multivariate regression confirmed that β-blocker use exerted no modifying effect on post-treatment improvements in VE/MVV ratio, peak VO_2_ or O_2_ pulse after 12 weeks of tiotropium/olodaterol therapy [75]. Collectively, CPET parameters including DLCO and the VE/VCO_2_ slope provide objective stratification evidence for β-blocker selection: carvedilol may be prioritized for HF patients with preserved diffusion capacity and marked exercise hyperventilation, while β_1_-selective blockers represent the safer preferred regimen in HF-COPD overlap patients vulnerable to impaired gas exchange and hypoxic stress [70].

## 8. Effects of Bronchodilators on CPET

Inhaled bronchodilators lead to consistent improvements in CPET-derived exercise parameters in patients with chronic obstructive pulmonary disease (COPD, including pre-COPD individuals with isolated air trapping), predominantly by alleviating static and dynamic hyperinflation rather than modifying physiological dead space fraction (VD/VT) during exertion. Fixed-dose LABA/LAMA dual combination therapy prolongs constant-work-rate exercise endurance by approximately 17% compared with a placebo; this functional benefit is driven by preserved inspiratory capacity throughout exercise without significant changes in isotime VD/VT, confirming relief of air trapping is the core therapeutic mechanism rather than improved ventilation–perfusion matching [76]. Even in COPD patients without baseline dynamic hyperinflation, combination bronchodilation still elevates resting inspiratory capacity, expanding ventilatory reserve and raising maximal exercise tolerance, while those presenting exercise-induced dynamic hyperinflation gain relief via blunted progressive end-expiratory lung volume elevation. Both subgroups exhibit comparable gains in endurance time post-treatment, indicating dual complementary pathways for functional improvement [77]. While the short-acting β_2_ agonist albuterol greatly improves V_E_/VCO_2_ at the peak of exercise, it does not yield uniform CPET benefits across all smoke-exposed individuals with preserved spirometry. Significant increases in peak VO_2_, tidal volume, and O_2_ pulse are only seen in highly adherent subgroups with severe air trapping (RV/TLC > 0.35), supporting the notion that stratification by lung volume indices is effective for screening individuals with favorable responses to inhaled bronchodilators [78]. From a clinical perspective, bronchodilator-induced reductions in thoracic hyperinflation also alleviate mechanical cardiac compression and mitigate exaggerated intrathoracic pressure swings, which partially correct the confounding respiratory factors that distort CPET and echocardiographic markers of diastolic function in HF-COPD overlap patients.

## 9. Conclusions and Limitations

Heart failure combined with COPD constitutes a common and complex disease state in clinical practice, and diagnosing and assessing it require comprehensive application of multiple examination methods. Cardiopulmonary exercise testing, as a non-invasive and comprehensive functional assessment tool, plays an indispensable role in differential diagnosis, prognostic evaluation, and therapeutic decision-making for these patients. Current evidence indicates that the VE intercept (AUC: 0.88), breathing reserve (AUC: 0.91) and OUES (AUC: 0.87) have great ability to discriminate between COPD and HF, while Peak VO_2_, OUES, VE/VCO_2_ slope, and circulatory power facilitate prognostic stratification and risk assessment. Integrated application of pulmonary function parameters and CPET enables comprehensive evaluation of the cardiopulmonary system, enhancing diagnostic accuracy for this complex patient population.

**Figure 1:**
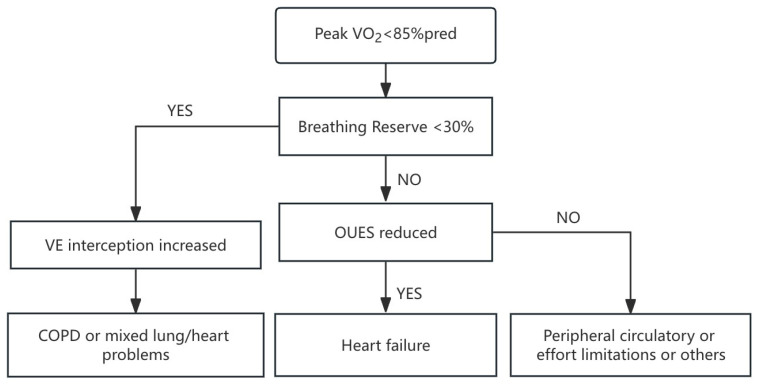
Tentative algorithm distinguishing ventilatory and cardiac exercise limitation.

Different overlap subtypes exhibit different CPET characteristics and clinical prognostic differences: HFrEF-COPD is characterized by severe circulatory function impairment and an increased risk of cardiovascular events, while HFpEF-COPD is more prone to respiratory function deterioration and acute exacerbations of COPD. Classifying COPD-HF overlap syndrome patients according to HF ejection fraction subtypes and their CPET profiles enables clinicians to move beyond the traditional “one-size-fits-all” management approach and formulate individualized treatment strategies targeting the dominant pathophysiological mechanism. CPET also provides a comprehensive prognostic stratification system for these high-risk populations, with both universal and subtype-specific high-risk markers that can help in predicting hospitalization and mortality risk. Figure 1 provides a potential algorithm to distinguish different patient groups via these CPET parameters.

Nevertheless, several noteworthy limitations constrain the clinical extrapolation of the conclusions summarized in this review. First, many critical CPET cut-off values referenced herein originate from single-center research with limited sample sizes, with no external multi-center validation, restricting their widespread use across general clinical cohorts. Second, all the CPET variables discussed are continuous rather than absolute binary thresholds. Advanced HFrEF-COPD and HFpEF-COPD overlap patients frequently exhibit concurrent severe ventilatory and circulatory defects. Clinicians must judge the primary exercise-limiting factor based on holistic clinical context instead of excessively relying on isolated numerical cut-offs. Third, concomitant COPD creates substantial diagnostic barriers for HFpEF identification [79]. Prior primary care data demonstrate poor-quality echocardiographic images in 10.4% of all COPD patients [80], rising to 35% in severe COPD [81] and 50% in very severe airflow obstruction [82]. Consequently, misclassification bias may have affected some previous CPET cohorts, where patients with isolated COPD complicated by secondary right ventricular dysfunction were misdiagnosed as having left-sided HFpEF. This inherent diagnostic limitation partially undermines the reliability and generalizability of the CPET phenotypic profiles summarized in this review. Comprehensive interpretation incorporating imaging findings, pulmonary function data and overall clinical status is mandatory when evaluating these parameters in routine clinical practice. Routine clinical interpretations of CPET parameters must integrate cardiac imaging, pulmonary function tests and full clinical background rather than relying on exercise data alone.

## 10. Future Directions

Despite current limitations in standardization, sample size and interventional evidence, CPET remains the core examination supporting precision management for COPD-heart failure overlap syndrome. Future research on CPET in the field of COPD-HF overlap syndrome should focus on addressing the current limitations and further improving the clinical application value and precision of CPET, with the following key research directions [36,53]. First, conduct large-scale, multi-center, international cohort studies to validate the subtype-specific CPET diagnostic cutoffs in diverse real-world populations, and establish standardized calculation methods and age, gender and ethnicity-stratified normative reference values for core parameters such as VE intercept and VE/VCO_2_ slope [36]. Second, explore the integration of novel biomarkers and monitoring technologies with CPET: near-infrared spectroscopy (NIRS) can be used to assess peripheral muscle tissue oxygenation, potentially enhancing the detection of early cardiac dysfunction in COPD patients and improving the diagnostic accuracy of occult HF-COPD overlap syndrome [53]. Third, develop artificial intelligence and machine learning models based on CPET data: integrate CPET waveform parameters, echocardiographic data, clinical characteristics and pulmonary function indicators to build automated differential diagnosis and risk stratification models, which can improve the objectivity and efficiency of CPET result interpretation and realize precision phenotyping of overlap syndrome [6]. Fourth, design and conduct CPET-guided interventional RCTs: enroll patients according to CPET subtypes (HFrEF-COPD vs. HFpEF-COPD) and compare the efficacy of subtype-specific treatment strategies with conventional treatment, to verify the clinical benefits of CPET-guided treatment in improving prognosis, reducing readmissions and mortality [53]. Fifth, develop simplified and portable point-of-care CPET devices to reduce the technical and equipment requirements of CPET, expand its accessibility in primary care and emergency settings, and enable early screening and diagnosis of overlap syndrome patients in the community [83].

## Data Availability

No new data were created or analyzed in this study. Data sharing is not applicable to this article.
